# Listen to people with cognitive impairments: Communication difficulties and strategies

**DOI:** 10.1093/geroni/igaf122.2581

**Published:** 2025-12-31

**Authors:** Hui-chuan Hsu, Shu-Nu Chang-Lee

**Affiliations:** Taipei Medical University, Taipei City, New Taipei, Taiwan (Republic of China); National Quemoy University, Jinning, Kinmen, Taiwan (Republic of China)

## Abstract

**Purpose:**

This study aimed to explore the communication skills and difficulties between people with different degrees of cognitive impairments and formal/informal caregivers to provide suggestions for effective communication for caregivers.

**Methods:**

We observed the interaction of older adults with cognitive impairment from a long-term care facility, a day-care center, and a community-based care center. Videos with 1 to 3 sessions recorded each older adult’s interactions with their formal or informal caregivers; each session was 10 to 20 minutes. In total, 25 older adults participated in this study. All the videos of dialogues, facial expressions, motion, and gestures in the interactions were coded as transcripts, and then the communication strategies, difficulties, and diagnoses were analyzed.

**Results:**

Three themes were categorized: (1) Communication strategies of caregivers: The most often used strategies of caregivers included repetition, rewording, extending questions, forming assumptions, providing possible answers, following the topic, self-sharing, inappropriate questions, asking but not listening, and using assistive tools. (2) Communication characteristics of older adults: Vocabulary difficulty, longer time to respond, incomplete sentences and hidden meaning, sharing life experience, health problems and illiteracy, changing subjects or pretending, silence or refusing to respond, inability to remember, and non-verbal language. (3) Skills to improve communication: Understanding their background and stories, conquering cohort differences, using the same language/dialects, questioning but not testing, listening and conversation extension, and being patient and sympathetic.

**Discussion:**

Caregivers’ communication strategies, strengths, and weaknesses were diagnosed. General communication principles and personalized suggestions are provided.

